# Opening up New Geographical Ontologies around Adapting to Climate Change

**DOI:** 10.1111/tesg.12552

**Published:** 2023-03-13

**Authors:** Susannah Fisher

**Affiliations:** ^1^ Geography Department King's College London London UK; ^2^ Institute for Risk and Disaster Reduction University College London London UK

**Keywords:** climate change, governance, regional, non‐state actors, adaptation

## Abstract

Opening up regional ontologies for climate action is a necessary and underexplored dimension of climate change policymaking. This commentary explores how a regional lens might be integrated into the complex mosaic of climate governance, particularly in the context of resilient regions. I argue regional ontologies for climate policymaking could have greater analytical power if integrated into a theoretical framing of action that goes beyond the nation‐state, beyond formal policy processes and beyond a strict binary between science and policy. Applying this lens to resilient regions, I argue there are particular opportunities at the regional scale for highlighting diverse perspectives or adaptation issues obscured through a national ontology, using existing transnational data infrastructure and community‐led data systems to support the regional ontology and reframing the scale of collective future visions for a climate‐adapted world.

In the UN climate negotiations at the end of 2022, national government representatives sat in deadlock in the beach resort of Sharm el‐Sheikh seeking to negotiate what a global target should be for adapting to climate change, known within the negotiations as the global goal on adaptation. Some Parties wanted the goal to contain targets and a framework of important dimensions. Others wanted to keep the negotiating text broad and spend more time developing the details. Many of the differences come down to differing national incentives for the goal and disagreements between countries over the role of climate finance in the process (Beauchamp & Motaroki 2022). In UN workshops throughout the year as well as in the IPCC Working Group II report released in February that year, technical challenges to assessing a global target were extensively discussed with multiple reports concluding there was insufficient data or understanding of effective adaptation to make a meaningful global assessment (AC 2022; IPCC 2022; UNEP 2022).

This tension in the ongoing negotiations highlights the impact of the ontological gap Taylor outlines in his article—*The geographical ontology challenge in attending to anthropogenic climate change: regional geography revisited* (2023)—the lack of a strong regional lens in climate action. When the primary unit for climate action and politics is understood as the nation‐state, then assessing collective progress becomes an aggregation of all nation‐state progress. In the case of the global goal on adaptation, this makes little sense in terms of advancing an adaptation agenda, increasing ambition or improving implementation. Aggregating progress made in the small island country of Tuvalu towards managing storm surges along the coast, advances in Australian wildfire management and outcomes of Kenyan food security programmes with activities funded through UN funds, multilateral banks and national budgets makes for a disparate collation of widely differing contexts, needs and priorities. One layer which could help address this is the level Taylor describes in his article: the region, understood as both formal and/or functional. For example, assessing progress across whole coastlines at risk from hurricanes across North America and the Caribbean, a river delta such as the Ganges or regional food supply chains, would open up new understandings of progress and potential avenues for action.

In this commentary, I take Taylor's useful proposition and extend the argument in two directions. Firstly, I explore how a regional ontology might be integrated into a broader understanding of the mosaic of climate action, and secondly, I develop these ideas in more detail through the proposition of resilient regions.

## REGIONAL ONTOLOGIES IN A MOSAIC OF CLIMATE ACTION

There are already some networks and new coalitions of actors shaping climate action outside the political ontology of nation‐states. For example, cities have formed peer‐to‐peer networks that are taking independent action on mitigating and adapting to climate change beyond their national policy commitments (Fisher 2014). Some countries are acting in regional political alliances on the global stage. Geographical literature has theorised this fragmented and polycentric picture as one of governance and identified a range of mechanisms shaping climate action beyond the formal state‐centric policymaking of the IPCC and the UNFCCC (see Andonova 2010; Bulkeley & Newell 2015; Jordan *et al*. 2015). Regions have an important—and often neglected—role to play within this landscape and understanding the extent of their influence requires ontological interventions to be integrated into this complex mosaic to offer the most analytical power. This goes beyond Taylor's proposition that the regional perspective will be added onto the national ontology. It requires the regional perspective to be integrated into an understanding of this wider governance mosaic of climate action involving state, non‐state and sub‐national actors. In the following section, I explore what this integration would look like through discussing the role of non‐state actors in shaping formal policymaking, the role of non‐state and sub‐national actors in shaping climate action, and the relationship between the formal institutions of science and policy around climate change.

Regional ontological interventions sit within a complex policy landscape where non‐state and sub‐national actors play significant roles in shaping outcomes within the UN regime through mechanisms such as advocacy, finance and technical support (Nasiritousi *et al*. 2016). The regional interventions would be another piece of a complex political puzzle offering varying degrees of authority in different contexts through new and possibly unexpected alliances. With the explicit recognition in the Paris Agreement that climate action goes beyond formal state action, it could be argued the ontology embedded in the multilateral system itself is moving towards a networked one with nation‐states being one site of climate policymaking within a broader collection of actors with varying responsibilities. For example, the recent Sharm el‐Sheikh Adaptation Agenda is one manifestation of this mosaic. In Sharm el‐Sheikh, the COP 27 Presidency announced the agenda containing outcome areas and aspirational targets for global progress on adaptation in partnership with a group of high‐level champions and a range of UN agencies. In an attempt to increase ambition, the Presidency sought to build a wider coalition of state and non‐state actors who could build momentum. Another development over COP 27 was the agreement to fund loss and damage from climate impacts and here again rather than being constrained to nation‐states as the primary suppliers of finance, the negotiating text refers to new and innovative sources of finance, suggesting the potential for a mosaic of solutions going beyond nation‐state actors. These could include taxes on fossil fuel companies, aviation or shipping. A regional intervention in these contexts would not only need to reconfigure nation‐state relationships but also engage with corporations, transnational organisations and civil society to shape and implement climate policies.

Non‐state and sub‐national actors also play a role in directly governing climate action outside of nation‐state policies and multilateral agreements through for example action to reduce greenhouse gas emissions within city networks, supply chain risk management by corporations and investments made by the finance sector in high‐risk locations. These mechanisms can also be indirect and include shifting norms and values, changing incentives and framing solutions and relevant knowledge (Merry 2016). International organisations are part of an influential knowledge economy shaping what sustainable development means and how it is measured, and this influences how development is understood in many contexts (Fukuda‐Parr *et al*. 2014; Stone 2019; Bandola‐Gill *et al*. 2022). This circulation of technical consultants, data standards, templates and guidelines shapes how climate action is understood and implemented (Gupta *et al*. 2012; Turnhout *et al*. 2014). Taylor argues that the current deeply embedded spatial ontology around climate action ‘ensures policymaking on global issues is channelled through states’ (p2). While this is the dominant ontology within the multilateral system, there are alternative ontologies around who or what shapes climate action. Taking a wider theoretical lens of governance rather than focusing only on formal policymaking opens up these new potential ontologies and puts in perspective the at times limited role that formal policymaking can play in these areas. It is therefore useful to expand Taylor's proposition to ask whose ontologies are embedded in different fora and what they highlight or obscure both for analysis and for climate action. Moving beyond the nation‐state ontology could lead to seeking action at the level of polluting industries regardless of national allegiances or seeking to shift risk behaviour through investment guidelines rather than through a formal COP decision. Some of these changes are already being seen. Corporate actors or networks of actors may increasingly be held responsible for the impacts of climate change, as shown in the number of climate litigation cases seeking damages from fossil fuel companies for changes in local livelihoods (Ganguly *et al*. 2018). The Bridgetown Initiative discussed at COP 27 seeks action at the level of global finance rather than nation‐states, recognising the power actors such as the World Bank have to shape responses to climate change. Regional ontological interventions will have more saliency if they deliberately use and build on all forms of governing, going beyond only formal policy arenas to domains that offer the most opportunity and space for new actors to catalyse transformative change.

It is also useful when considering ontological interventions to conceptualise the relationship between the scientific institution of the IPCC and the political one of the UNFCCC as a negotiated and contested line between science and policy (Mahony & Hulme 2018). Boundary work along this line is ongoing and has included spatial dimensions when negotiating epistemic diversity, fair representation and the role of national governments (Beck & Mahony 2018). Beck and Mahoney argue that the IPCC assessments have the potential ‘to shape fields of political possibility’ and this offers a way into the ontological interventions discussed by Taylor. Understanding the IPCC as not only a site of scientific production, but also a site for contesting ontologies illustrates some of the ongoing debates on the nation‐state framing and potential leverage points for change. It provokes questions such as what would scientific inputs look like for regional ontologies and how would the defining power of nation‐states be managed to allow differing ontologies to emerge? The latest Working Group II report on Impacts, Adaptation and Vulnerability organises its analysis across ecosystems and different climate risks so offers a model that goes beyond the national ontologies. There is little evidence of those categorisations becoming embedded beyond the scientific community as political domains, for example, but it offers a potential set of new alliances that could be developed. One example of how these alliances might operate is the Alliance of Small Island States (AOSIS)—covering 39 small island and low‐lying coastal developing countries. AOSIS is able to amplify the specific needs of this group and gain greater traction for island issues in global fora (Ourbak & Magnan 2018). However, AOSIS is still built on a nation‐state ontology, going beyond this with a regional ontological intervention could incorporate small islands from other nation‐states, whose issues may not gain much saliency in their (predominantly non‐island) national politics.

In this first section, I have argued that we can take the proposition of regional ontologies for climate policymaking further by integrating these interventions into the complex mosaic of climate action that goes beyond the nation‐state, beyond formal policy processes and beyond a strict binary between science and policy. The regional lens is underexplored in these contexts and has much to add. Situating it within this mosaic allows a nuanced analysis of how regional ontologies would operate within this polycentric picture, the relationship they could have to the non‐state and sub‐national actors as well as the role they might play in changing norms, values, scientific cultures and shaping the science–policy interface.

## GOING FURTHER ON RESILIENT REGIONS

The second area where I wish to extend Taylor's arguments is around what new geographical ontologies for resilient regions might look like. Starting from Scott's argument (1998) that the state is unable to address complex issues and the common challenges in inter‐state relationships described through International Relations, I argue there is a need not only to find new groupings of states and regional institutions as Taylor argues but also to use new structures or incentivise different types of relationships that are more inherently suited to the complexities of decision‐making around adapting to climate risks. Adapting to climate impacts may involve trade‐offs between different agendas and priorities, there will be winners and losers and action needs to prevent maladaptation in surrounding areas or across wider networks and flows (Schipper 2020). While overlapping policy jurisdictions is one place to start, to address these complex and often conflictual issues will need new forms of decision‐making to move beyond the challenges experienced within a national ontological framing. Adaptation so far has been incremental and lacked the systemic changes needed to address the fundamental causes of vulnerability (Berrang‐Ford *et al*. 2021). In urban contexts, complex and overlapping jurisdictions where responsibility and agency are diffuse have contributed to a lack of adaptation action (Fisher & Dodman 2019). One way to move past this tension, is to conceptualise the role of the resilient region beyond just formal policymaking, to be one of building shared norms, values, communities and political constituencies around different approaches to risk management through connecting diverse actors. Over time, these new constituencies may gain political or economic power, directly access climate finance to implement their own interventions or reshape national politics around different forms of risk.

Building on Taylor's ‘relatively modest’ regional governance proposals for areas of risk, I argue there are other roles that could enhance the function of resilient regions. Firstly, there are issues of climate impacts that are hidden at the national scale but may not become fully visible if a regional focus does not explicitly move beyond an aggregated understanding of national risks. National ontologies may underplay the role of transboundary risks and global policy issues such as international migration and trade that require action at a different scale. A regional ontology could also make visible risks that are not prioritised by any powerful nation‐state, but when aggregated across regional contexts have a global impact such as sub‐national islands. Some risks may only become relevant when multiple shocks are experienced simultaneously that may not be fully considered in any one national plan, or a regional lens may show negative effects of a national project in neighbouring countries through the redistribution of risk.

A second dimension to consider in a resilient region that was more than a collection of nation‐states would be data architecture, and how the region would ‘come to be known’ when many data systems rely on national structures which underpin and reinforce a national ontology. Here, I suggest the transnational data systems of international organisations have a role to play. Tools such as the multi‐dimensional poverty index can be broken down to understand risks among different groups and offer comparable methodologies combined with some data infrastructure through the international system. The World Bank and UN statistical offices support a range of surveys that are increasing standardisation across national contexts and could also speak to a regional ontology. Another space for data framed with a regional ontology could be locally generated data, for example, the data collection by residents from informal settlements using settlement profiles, house‐by‐house surveys and mapping supported by Slum and Shack Dwellers International (Patel *et al*. 2012). These local data collection efforts are tools for political advocacy and ‘make visible’ communities living in informal settlements often missing from formal mapping exercises and censuses undertaken by the nation‐state. These can be combined into international or regional platforms with standardisation and verification (Beukes 2015). This also shows the possibility of the regional ontology in giving space and visibility to populations that are obscured within national boundaries.

Thirdly, a regional ontology could shape how and at what scale visions of the future are built and sustained for living with climate impacts. Adaptation policies and programmes embed an implicit or explicit framing of the type of future a country is seeking to move towards. In the current dominant ontology of adaptation that visioning process remains at the level of the national government, although there are varying levels of engagement at local scales. These ideas can become embedded as collectively held visions or socio‐technical imaginaries (Sismondo 2020). These imaginaries can shape national policy choices around science and technology and become entrenched in institutions, narratives and public modes of reasoning. The imaginaries are not necessarily immutable but can be contested over time through deliberate challenge or through the day‐to‐day juxtaposition of alternative visions or approaches. The future contained in adaptation plans reflects the future imagination of certain actors. The aspirations of marginalised groups or constituencies may not find space within national ontologies and a regional intervention could support these groups to imagine their future in a regional framework in ways that gain traction and support. A regional ontology could build future‐orientated constituencies beyond an aggregation of nation‐states and national politics that can be exclusionary, connecting regional communities with similar challenges in new ways. This could include building a collective vision among communities of forest‐dwellers in the Amazon or those living in the drylands in West Africa.

To conclude, I argue that situating the resilient regions within the mosaic of climate action discussed above, offers new points for a regional ontological intervention to shape action in new ways. The values and norms shared within the transnational community of international organisations and multilateral banks, for example, may shape national action through lending practices, understandings of credibility and expertise and technical assistance that supports a particular framing of adaptation (Singh *et al*. 2021). As a regional ontology becomes stronger, regions could develop their own institutional cultures, heuristics and knowledge practices that support the epistemologies and priorities of the region and govern action in new ways. The regions could also go beyond groupings of national governments and give political space to constituencies such as young people, those living in certain ecosystems, cities and/or non‐governmental organisations. This would mirror and support the polycentric governance model of the Paris Agreement, using a regional ontological intervention to uncover perspectives, data and governance mechanisms usually obscured within a dominant national ontology.
